# Genomics and Insurance in the United Kingdom: Increasing Complexity and Emerging Challenges

**DOI:** 10.1017/S1744133124000070

**Published:** 2024-05-16

**Authors:** Padraig Dixon, Rachel H. Horton, William G. Newman, John H. McDermott, Anneke Lucassen

**Affiliations:** 1 Nuffield Department of Primary Care Health Sciences, University of Oxford; 2 Centre for Personalised Medicine, University of Oxford; 3 Clinical Ethics, Law and Society research group, Wellcome Centre for Human Genetics, University of Oxford; 4 Evolution, Infection and Genomics, School of Biological Sciences, University of Manchester; 5 Manchester Centre for Genomic Medicine, Manchester University NHS Foundation Trust, Manchester

## Abstract

This article identifies issues relating to the use of genetics and genomics in risk-rated insurance that may challenge existing regulatory models in the UK and elsewhere. We discuss three core issues: (1) As genomic testing advances, and results are increasingly relevant to guide healthcare across an individual’s lifetime, the distinction between diagnostic and predictive testing that the current UK insurance code relies on becomes increasingly blurred (2) The emerging category of pharmacogenetic tests that are predictive only the in the context of a specific prescribing moment (3) The increasing availability and affordability of polygenic scores that are neither clearly diagnostic nor highly predictive, but which nonetheless might have incremental value for insurance underwriting beyond conventional factors. We suggest a deliberative approach is required to establish when and how genetic information can be used in risk-rated insurance.

## 1 Introduction

This article identifies emerging issues relating to the use of genomics and genetics^1^ in insurance. These issues relate to clinical, research, technological, and economic developments that may disrupt prevailing regulatory models intended to support the efficient and equitable provision of insurance. Our focus is on the specific regulatory context of the United Kingdom, but the general issues we raise will apply to varying extents in other jurisdictions.

Diagnostic tests for genetic conditions began to be used by the UK insurance industry to inform offers of cover in the 1990s, in light of which a “Genetic Testing Code of Practice” was introduced by the Association of British Insurers (ABI) in December 1997 (House of Commons Science and Technology Committee 2001). A version of this agreement has existed in various forms ever since and has been subject to amendments over time (Department of Halth and Social Care 2022). The current version of the Code, a voluntary agreement between the UK government and the ABI, refers to two types of genetic test.

*Diagnostic* genetic tests are defined as those that confirm or rule out a diagnosis. *Predictive* genetic tests assess future disease risk. These tests were first offered in the context of a previously identified familial “genetic disorder” to see whether an individual might develop the condition in the future. They were therefore typically only offered to people with a personal or family history that strongly suggested a genetic condition. In this scenario, if a potentially concerning variant was identified there was a much higher probability of it being medically relevant, permitting greater confidence in diagnosis.

The Code applies to life, critical illness, and income protection insurance, although we consider issues affecting other forms of insurance below in the context of emerging challenges that may go beyond the current version of the Code, or which may arise in other countries. The Code embodies two core principles. The first prohibits insurers from requiring or pressuring applicants for insurance policies to take either type of genetic test. The second relates to predictive tests. While the Code permits consideration by insurers of diagnostic genetic test results, predictive tests results may be considered only if the test is specifically named in the Code and if the financial sum to be assured exceeds limits defined in the Code. Only one such test currently meets these criteria, which is a predictive genetic test for Huntington’s disease in relation to applications for life insurance cover over £500,000. The Code does not prevent the use of family history for a disease or trait. Strongly heritable conditions will sometimes have a family history and insurers can and do use this information as an indirect genetic test of disease risk.

A long-standing concern (Prince 2018, 2017, Prince et al. 2021, Ossa and Towse 2004, Holmes 1996, Rothstein 2018, Daniels 2004, Joly, Dalpé, et al. 2020, Joly, Dupras, et al. 2020, Tiller et al. 2022) that motivated the original version of the code and its subsequent iterations has been the possibility of genetic discrimination, which could involve the denial of insurance, restrictions to coverage, or substantially higher premiums to those with particular genetic profiles (Holmes 1996, Harper 1992, Maxwell et al. 2021). This could lead to individuals refusing to take genetic tests that were otherwise indicated for fear that their results (or the mere fact of taking the test) could result in exclusion or unfavourable terms when seeking insurance. On the other side of the market, prohibitions or limitations on the ability of insurers to use genetic information in risk-based pricing could threaten their commercial viability or lead to the withdrawal of particular insurance products (Born 2019, Howard 2014).

These types of concern were, in some cases, motivated by an expectation that most genetic tests would be highly predictive, leading to this testing being potentially discriminating (Macdonald and Yu 2011). However, although many genetic traits are highly penetrant, a large proportion are not, meaning that testing for these genetic variants in unaffected individuals may not be as predictive as once imagined. To date, evidence from annual reports on the operation of the Code (ABI 2022) suggest genetic information on insurance been limited in all but a few cases. However, these reports cannot determine how many people have elected not to declare genetic risks (since are not obliged to do so), and therefore do not necessarily fully reflect the impact of these arrangements on insurance decisions. In any event, advances in technology that enable the identification of more subtle genetic contributions to disease susceptibility, longevity, and drug responses, may merit new forms of oversight to support the interests of both insurers and their policyholders (Rothstein 2018, Tiller et al. 2020, Born 2019, Roberts, Dolinoy, and Tarini 2014, Conley 2019, Peter, Richter, and Thistle 2017, Rodriguez-Rincon et al. 2022).

We describe three developments that may increase the salience of genetic data for insurance. The first issue relations to a blurring of the distinction between diagnostic and predictive genetic tests, the second to similar issues in the context of pharmacogenetics, and the third to the prediction of healthcare costs, mortality and related phenotypes. We discuss these three issues below after first briefly reviewing the principles of insurance in the context of actuarial fairness and wider considerations (beyond actuarial fairness) that may have a bearing on how genetic tests might be used in insurance.

## 2 Insurance, actuarial fairness, and wider considerations regarding genomics

Insurance protects against losses associated with unpredictable events. While an event may be probable (such as some form of prolonged ill health) or certain (death), its timing and consequences are likely to be unpredictable. Faced with uncertainty about the timing, scope and extent of these events, individuals derive value from pooling risks with others in the population. Within a risk pool, the majority of individuals who do not make claims contribute to meet the cost of the minority who do make claims. Our discussion of insurance in this paper refers throughout to risk-rated insurance, as opposed to for example, community-rated insurance which is a method of determining insurance premiums based on the overall risk profile of a community or group rather than individual characteristics.

The premium and terms of risk-rated insurance contracts reflect the risk that a prospective customer may experience an event that gives rise to a claim, as well as the costs borne by the insurer in providing cover. Higher assessed risk generally results in higher premiums to be paid by customers, and/or more restrictive contract terms. The converse will generally be true for lower assessed risk. From an actuarial perspective, a fair insurance contract is one that accurately prices risk. Systematic mispricing of insurance by a single provider, in the sense of overcharging or undercharging certain groups given the risks and therefore the costs associated with each group, will result in a competitive disadvantage and will not be sustainable.

If genetic factors, broadly defined, influence the risk of insurable events, then their use in insurance underwriting will contribute to actuarial fairness in the pricing of risk, and the efficient operation of the insurance market as a whole. Reliance on actuarial fairness “expresses the moral judgment that fair underwriting practices must reflect the division of people according to actuarially accurate determination of their risks” (Daniels 2004). Wider considerations in relation to the use of genetics in insurance beyond actuarial concerns may involve access to insurance by different groups, the cost and quality of insurance, and privacy issues. These wider considerations motivate the existence of the Code, as well as other international examples that treat genetic information differently to other rating factors used in insurance underwriting.

These international examples include the Genetic Information Nondiscrimination Act (GINA) in the United States (Bélisle-Pipon et al. 2019), which prohibits the use of genetic information in determining the offer of health insurance, but not necessarily other forms of insurance including life insurance. The Genetic Non-Discrimination Act (Bombard and Heim-Myers 2018, Bélisle-Pipon et al. 2019) (Supreme Court of Canada 2020) in Canada prohibits requesting disclosure of the results of genetic tests or being forced to take such tests in order to obtain access to goods and services including insurance. The Australian life insurance industry introduced a partial, self-regulated ban on the use of genetic results in 2019, and debates continue on whether this moratorium is fit for purpose (Tiller et al. 2023, Tiller and Lacaze 2023).

As we assess emerging challenges posed by genomics to insurance, and specifically in the regulatory context of the United Kingdom, we consider challenges both to the process of underwriting (“how much risk is attributable to a particular person?”), to these wider considerations that may give rise to departures from actuarial fairness in offers of insurance, and finally to the operation of the insurance market itself. We examine these challenges under three primary themes, as follows.

The first relates to the fact that, as genetic testing routinely encompasses ever greater portions of the genome, the distinction in the Code between diagnostic and predictive genetic testing becomes blurred. A “typical” person has around 100,000 rare variants in their genome (Auton et al. 2015) - some of these may help diagnose a condition already known about, others may predict disease (with varying degrees of accuracy) and yet others be entirely uncertain as to what their effects may be. This blurs the distinction alluded to in the Code. This blurring may also increase the challenges of underwriting (given uncertainty associated with the interpretation of results), with wider considerations relating to the equitable processing of this information, and with the wider operation of the market (given the resources necessary to process increasing volumes of genetic data).

The second area relates to pharmacogenetics, which is the study of how genetic variation can affect an individual’s response to medicines. However, predictions of response at an individual level are imperfect, the medicine in question may never be required, and identification of the risk of serious adverse events could increase the cost of future healthcare and therefore potentially the costs of insurance (if no effective alternative treatments are available) or reduce these costs (if treatment is more effective when informed by pharmacogenetics). Again, pharmacogenetics seems likely to blur the diagnostic/predictive distinction at the centre of the Code, and will likely have implications for underwriting and for equitable access to insurance.

The third area relates to predicting costs, mortality, and related phenotypes from genotype, especially in the context of increasing availability and affordability of composite indices of disease liability such as “polygenic scores” that measure a component of risk for common disease. These tests are neither clearly diagnostic nor highly predictive, and the contribution of a polygenic score to absolute risk may be very small. Nonetheless, polygenic scores could add some value to underwriting beyond conventional factors, which, absent other considerations, would result in better pricing of risk. However, it is these other considerations that merit a wider debate on appropriate uses of this type of information, even if these scores are only weakly predictive of risk. There may also be market-wide impacts under differential access to this information.

We explore each of these topics in more detail in the following sections.

## 3 The blurring of the distinction between diagnostic and predictive tests

The cost of genetic testing continues to fall, and the volume of data that such testing produces continues to increase (Horton and Lucassen 2019). Whilst in the past only certain variants were analysed based on a clinical suspicion of their presence, “genome first” approaches facilitated by technological advances identify many more variants that may have implications for the individual concerned, and interpreting their significance can be very challenging. To place the scale of variation in context, there are on average 4-5 million differences between the reference human genome and any typical human genome (Auton et al. 2015) – and many of these differences will have minor or unknown medical impact.

Indeed, large numbers of variants that have historically been considered to be pathogenic (that is, associated with specific health outcomes) have in fact turned out to be common in individuals who do not show the associated phenotype, suggesting that either their original classification was wrong, or that their impact on health is more subtle or context-dependent than previously appreciated.

Beaumont and Wright (Beaumont and Wright 2022) illustrated the challenge of interpreting people’s genomic data, showing that while large gene panels may maximize diagnostic yield, they are also likely to identify several variants that look hypothetically concerning though are probably benign; most people have at least one rare variant in the coding regions of the genome in panels containing over five hundred disease genes. Even for ‘well-understood’ pathogenic genetic variants, context matters: Jackson et al (Jackson et al. 2022) found that people with cancer-predisposing genetic variants were at significantly less elevated risk of cancer in the absence of a family history. These issues are likely to be amplified by initiatives to undertake whole genome sequencing of all newborn children within a population.

The predictive value of a specific variant identified via genetic testing in the absence of phenotype and positive family history may therefore be low (Horton and Lucassen 2019, Horton et al. 2019, Horton and Lucassen 2022). As genetic tests become broader, a distinction has emerged between using genetic results for diagnoses (in tandem with other clinical information) and the use of genetic data for other purposes (such as prediction of disease risk) outside of a clear familial or phenotypic context. For example, in the former case, a high degree of confidence might be expected in reaching an overall diagnostic assessment for a particular individual. In the latter, inferences about disease risk are likely to be less meaningful at the level of the individual.

This changing distinction between diagnostic and predictive results may also be influenced in some contexts by prognostic information. For example, in some cases an underlying genetic cause for a clinical diagnosis may change the prognosis associated with a particular condition. For example, knowledge that an individual has congenital long QT syndrome (which is associated with irregular or abnormal heart beats) may change the prognosis associated with the risk of future cardiac arrests (Arthur, Ahmad, and Pieter 2022).

There is also increasing interest in (Sud et al. 2023) aggregated summaries (typically referred to as polygenic risk scores) analyzing many points of variation in the genome to estimate liability to disease incidence, disease progression or related outcomes. Does knowledge that an individual has high polygenic risk for a particular condition constitute a genetic “result”? Without additional information, knowledge that an individual is in the top decile or even the top percentile of a polygenic distribution for incident disease may indicate only a marginal increase in lifetime risk, and furthermore could miss most cases that occur in people in other centiles.

For example, women in the top 5% of polygenic risk for ovarian cancer have a lifetime risk of 2.1% for developing this condition, compared a population average risk of 1.6% (Sud, Turnbull, and Houlston 2021). The overall distribution of the polygenic risk at the population level may be informative for disease etiology, even if knowledge of an individual’s polygenic risk in itself does not contribute much, if anything, to knowledge of “which particular individuals will succumb” (Smith 2011) to the condition of interest. Below, we explore other potential consequences of polygenic scores for insurance in the context of increasing knowledge of the association between genotype and healthcare costs.

These discussions illustrate that the distinction between predictive and diagnostic codes in the Code, based primarily on the presence or absence of symptoms, may be increasingly difficult to defend since this binary distinction may not be especially relevant to the insurance decision. Of note in this regard is that the UK Government opened a consultation in the second half of 2023 on whether (amongst other issues for which evidence was requested) the Code should widen the considerations used to characterize whether a predictive test should be disclosed. The consultation was structured around four questions: 1. How useful is the genetic test for characterizing the risk of developing a condition? 2. How many people take the test? 3. What is the impact of the condition in terms of the length and quality of life of people who develop it? 4. What is the potential for reducing the risk of developing the condition and managing its effects if it develops?

We note that these questions highlight a recognition that topics such as penetrance need consideration (since the “usefulness” of a genetic test in characterizing disease risk is a function of penetrance) and that this moves away from the symptomatic/asymptomatic distinction that is central to the current Code. For example, highly penetrant risk-increasing BRCA variants are predictive of future risk and may have material consequences for future healthcare costs (of interest to a health insurer) and for mortality (of interest to a life insurer).

### Insurance industry perspectives

Even if genetic variants found by genomic tests can - in certain contexts- help predict disease, one may argue that no changes to current practices are required. This would be the case if, in fairly assessing risks, insurers recognize that many apparently pathogenic genetic variants may in fact turn out to be clinically insignificant, and as such avoid gross distortions to the pricing of risk. However, the situation may be more complicated than this given the responses that insurers and individuals may make to genetic information. Lacaze et al (Lacaze et al. 2017) note that this depends on the insurer “actually understanding” the genetic concepts involved in influencing risk. Ashcroft (Ashcroft 2007) refers to irrational discrimination by insurers arising from “false beliefs” about genetic information. In this case, the identification of a variant, or a combination of variants, may at best lead to mispricing of the associated risk, or at worst an actuarially unjustified refusal to insure an individual. In either case, there would be too little insurance offered at too high a price.

For example, pathogenic variants in *MYBPC3* gene increase the risk of developing hypertrophic cardiomyopathy, a disease caused by dysfunction in the cardiac muscles which can lead to arrhythmia and sudden death (Marston et al. 2012). Many people, even within the same family, with the same pathogenic *MYBPC3* variant may have no or few symptoms, will never develop hypertrophic cardiomyopathy and are unaware that they have this genotype (Marian and Braunwald 2017). These individuals are at increased risk of developing hypertrophic cardiomyopathy but the genetic test result itself does not mean they have the condition. Nevertheless, some individuals with these variants (and not necessarily expressing the associated phenotype) are reported to have encountered actuarially unjustified obstacles when applying for insurance (Christiaans et al. 2010).

These possibilities and concerns are also informed by historical precedents for these practices in relation to new and complex health challenges such as the emergence of HIV. For example, following guidelines issued in 1987 by the Association of British Insurers, applicants for life insurance were asked if they had taken an HIV antibody test (Barton and Roth 1992). This led to concerns that merely taking the test would lead to insurance being withheld or becoming more expensive, and moreover could lead to serious health consequences by deterring people from HIV testing, leading to increased rates of HIV transmission (Barton and Roth 1992).

### Consumer perspectives

A further consideration relates to the responses of individuals, and potential excessive insurance against negligible risks associated with variants that could theoretically increase the risk of developing a given phenotype but which ultimately are likely to be clinically insignificant. As McLean and Gannon (McLean and Gannon 1998) put it, “…the aura of scientific certainty which pervades much of the discourse on genetics may lead the unwary or the ignorant into weighing genetic evidence more heavily in the decision-making scales than is actually merited”.

Viewed from one perspective, this may not require policy intervention if individuals are considered to be the most competent judges of their own self-interest. However, concerns about the consequences of exaggerated expectations regarding the informativeness of genetic tests for future disease risk, such as increased, but unwarranted, demand on primary care, may provide a rationale for intervention. On the other hand, there is evidence that some at-risk individuals refuse or are inclined to refuse genetic testing from fear of discrimination by insurers (Hall and Rich 2000, Allain, Friedman, and Senter 2012, Haga et al. 2013, Wauters and Van Hoyweghen 2016, Robinson et al. 2016).

### Market and system-level perspectives

The resources needed to confirm the consequences for an individual of a particular genetic variant or variants may be considerable, and in some cases there will be no known consequences for an individual’s health. These resources, all with competing alternative uses, include patient time, clinical input, and potentially also financial consequences for the individual as well as the health system concerned. For example, McGurk et al (McGurk et al. 2022) considered recommendations for reporting of secondary findings in clinical sequencing following the list of secondary findings noted by the American College of Medical Genetics and Genomics that should be sought routinely. One example is the TTN gene; particular variants in this gene predispose to dilated cardiomyopathy (DCM), yet the lifetime risk of DCM for those with this variant is low. Some 8,000 person years of surveillance (amounting to 1,600 cardiovascular magnetic resonance scans under a 5-yearly imaging schedule) in the UK Biobank population (a cohort of middle-aged and early-old age individuals with the approximate age for presentation of dilated cardiomyopathy) are necessary to prevent one death over the subsequent four years. The yields of one-off and serial evaluation might be expected to be lower in younger individuals.

## 4 Pharmacogenetics

The complexities in defining which results merit intervention, and when, also arise in the context of pharmacogenetics. Response to medicines vary between individuals, in part, because of genetic variation. If a pharmacogenetic test determines that an individual is less likely to respond to a certain medication, it may be recommended to select an alternative treatment (if available). However, this alternative medicine may, on average in the population, be less effective than the “first line” therapy. Although this individual is receiving the most suitable treatment for them, it remains an inferior therapeutic strategy compared to what an individual without the pharmacogenetic variant would receive. Alongside the health consequences for the individual concerned, this has implications for potential future treatment costs. Pharmacogenetic testing may predict treatment options to some extent and confer an increased or decreased chance of response to therapy, but such findings are not usually thought of as diagnostic, again highlighting that the diagnostic/predictive classification may be less useful in this setting.

For example, *CYP2C19* is an important drug metabolizing enzyme (Gaedigk et al. 2017). Genetic variation in *CYP2C19* is associated with diminished tolerance, treatment failure, and adverse reactions for many medicines (Botton et al. 2021). For instance, *CYP2C19* catalyzes the activation of clopidogrel, a widely prescribed anti-platelet drug. Individuals with two *CYP2C19* loss-of-function alleles (“poor metabolizers”) will respond less well to clopidogrel compared to the rest of the population (Scott et al. 2013).

A genetic result showing that an individual carries a loss of function variant in *CYP2C19* means that their CYP2C19 enzyme will have reduced activity. As such, *CYP2C19* genotyping could be considered diagnostic in nature. However, the negative clinical impact of being a CYP2C19 poor metabolizer is only experienced in certain contexts, such as when the individual is prescribed clopidogrel or another antiplatelet medicine. In that regard, one could consider the test to be more akin to a predictive test. This blurring between the diagnostic and predictive creates challenges when attempting to consider the insurance implications of a given pharmacogenetic test result.

### Insurance, consumer, and market perspectives

We consider that a greater use of pharmacogenetic testing will further blur the distinction between diagnostic and predictive tests. The actuarial implications of pharmacogenetic drug responsiveness will be difficult to assess, not least because prescribing decision for these kinds of medication will arise for some but not all potential patients. Since improved prescribing would very likely improve patient health in aggregate, it is important that any guidance, regulations, or legislation support appropriate prescribing.

## 5 Predicting costs, mortality, and related phenotypes from genotype

Genetic rating factors that influence the propensity to incur healthcare costs and that influence mortality will be relevant to the actuarial pricing of health and life insurance products. We consider whether these kinds of rating factors might be feasible, and if so the types of wider considerations that merit scrutiny around their use.

Recent evidence has quantified the heritability of future healthcare costs. Heritability refers the proportion of variance in a phenotype that is attributable to genetic variance in a given population. Lakhani et al (Lakhani et al. 2019) used an American health insurance dataset to examine the heritability of monthly healthcare cost amongst 56,396 twin pairs. They estimated that the heritability of average monthly cost was 0.29, meaning that 29% of the variance in average monthly healthcare cost between individuals in that study population was attributable to genetic factors. de Zeeuw et al (de Zeeuw et al. 2021) studied 16,726 participants in the Netherlands Twin Register and estimated similar heritabilities between 0.29 and 0.38. Although debates continue about the extent to which twin studies might over-estimate heritability (Young 2019), these estimates for healthcare cost heritability suggest measures of genetic liability to incur healthcare costs are potentially relevant to insurance underwriting.

Mendelian randomization analyses – the use of common genetic variation indicating liability to particular phenotypes in causal instrumental variable analyses – has demonstrated that genotypes associated with a variety of diseases (Dixon, Harrison, et al. 2022), traits (Dixon et al. 2020, Hazewinkel et al. 2022, Lee et al. 2022) and behaviours (Dixon, Sallis, et al. 2022) are also associated with healthcare costs and with closely related outcomes such as rates of inpatient hospital admission (Hazewinkel et al. 2022). The use of cost phenotypes will be more consequential in health systems that rely on private healthcare insurance, and are unlikely to have a significant impact in tax-payer funded, universal and free-at-delivery health system such as the NHS in the UK.

However, these types of consideration may be important for life insurance in many countries. In relation to mortality risk, Linner and Koellinger (Karlsson Linnér and Koellinger 2022) found that a polygenic score could detect a substantially shorter median lifespan in the top decile of total genetic liability independent of other factors used in conventional insurance underwriting. There are emerging examples (such as(Insurance Newsnet 2023)) of life insurers using polygenic risk scores for health conditions (rather than mortality risk per se) as a means of encouraging behavioral change such as improved adherence to medication in light of personal knowledge of a polygenic risk.

### Insurance, consumer, and market perspectives

While polygenic risk scores for factors used in insurance underwriting may be neither particularly predictive nor diagnostic, the use of this kind of information could improve the actuarial fairness of assessed risk in offers of insurance. However, wider debates are needed on the normative implications of using genetic information on this way – this is the topic of our next section (“Toward deliberative processes”). A particular issue that may arise in this context is adverse selection.

Unlike in most other markets, the likelihood of incurring significant cost in the process of providing the service of insurance depends in a fundamental way on the unobservable characteristics of the buyer and the unobservable actions this buyer might take (Cutler and Zeckhauser 2000). Adverse selection arises in circumstances where individuals who expect to incur high future health costs differentially prefer more generous or comprehensive insurance plans, and individuals who expect relatively low costs select less comprehensive and less expensive plans (Cutler and Zeckhauser 1998).

One means to overcome adverse selection is to reduce the informational asymmetry between customer and insurer so that the latter can more readily identify risks, and offer contracts priced according to individual risk profiles This, of course, may conflict with the wider concerns and priorities concerning insurance in the presence of more extensive and richer genetic data than the insurance industry has heretofore encountered.

The magnitude of adverse selection informed by knowledge of polygenic risk in relation to longevity or future healthcare costs remains to be assessed, and may be small in general (MacMinn, Brockett, and Raeburn 2007) or large for some groups (Hoy and Witt 2007, Howard 2014). Overall, there remains little evidence on whether these concerns are having or will have noticeable impacts on the insurance markets concerned. However, given the emerging developments we describe above, there appears to be a strong case for considering whether new arrangements for oversight are merited.

## 6 Toward deliberative processes

The foregoing considered issues that were specific to the regulatory regime of the UK (specifically the Code), as well as more general issues that may arise both in the UK and elsewhere. A fundamental issue underlying both the specific and general issues to which these emerging developments in genomics give rise for insurance relates to whether and how genetic information merits distinct treatment as a risk factor for risk-rated insurance. The complexities include the uneven way in which genetic information may be revealed (and potentially disclosed) across types of individual, diseases, risk levels, and at points in the life course.

A central task that for any new regulatory response that involves wider considerations than the actuarial pricing of genetic risk will therefore be to identify individuals and groups on whom these costs fall and on whom they ought to fall. We do not take a position on which normative perspectives are necessarily appropriate, but instead outline the parameters in which such a debate may be held.

For example, normative considerations might suggest people at risk of some types of condition ought not to lose access to insurance or to face higher premiums because of their genetic risk. A *per se* rejection of risk-based pricing for these individuals shifts their costs onto those with lower (genetic) risk, with the effect that a cross-subsidy is created from lower to higher-risk individuals. This cross-subsidy may be very modest in scale, although the scale of this impact remains to be determined and overall consequence of rejecting risk-based pricing is unlikely to “net off” to zero costs. For example, depending on the nature of the risks and proportions of people in each risk category, it is possible (if not likely) that the reduction in costs for high risks is not as great as the increase in costs for the low risks. In this scenario, the aggregate costs of insurance become higher for society albeit the extent of increase remain to be established. Moreover, any potential gains from actuarial pricing of genetic risk (such as improved medication adherence incentivized by life insurance policies that request information on polygenic risk) would not be realised.

The risks faced by individuals are not abolished simply because some groups do not face actuarially fair risk-based pricing for their insurance products; instead, there will necessarily be impacts on the price and availability of insurance. There may be second order effects on the dynamics of competition amongst insurance. For example, adverse selection is still possible even if those with the highest genetic risk do not face premiums based on their actuarial risk. Higher prices amongst the lower risk groups may reduce their use of insurance, resulting in future avoidable health impairments and economic detriment. On the other hand, appropriate regulatory protections could prevent individuals being penalized from undertaking genetic testing where indicated (Filipova-Neumann and Hoy 2014).

The analytic challenges therefore involve characterizing and modelling the trade-offs involved (Wilson 2006, Ossa and Towse 2004). These trade-offs include the quantitative impact of genetic information on the terms and price of insurance, increasing knowledge of genetic contributions to disease and mortality, the predictive capacity of polygenic and other genetic risk scores, the demand for insurance, and the behavioural responses of consumers. If genetic data cannot be used in risk based-pricing, then variables correlated with these data could potentially be used (if available) and this may frustrate to some degree restrictions on the use of genetic data (Aseervatham, Lex, and Spindler 2016, Pope and Sydnor 2011). The feasibility of finding robust proxies for genetic data may be more limited than for more traditional risk factors such as gender (Oxera 2010) but this is likely to vary by context.

The normative issues require, we suggest, a deliberative approach to identify what deviations from actuarially fair pricing are appropriate. The final product of a deliberative process is “guidance shaped by judgements” (Culyer 2006). What judgements matter or ought to matter in this context? A fundamental consideration is to establish which conditions and which genetic risks (and their consequences) society assesses should be borne by the individual and which should be shared more widely.

A starting point is perhaps to recognize the value of insurance, and the need to ensure its sustainable provision. An overarching approach could be to minimize the overall cost of insurance, subject to specific exemptions the costs of which are either managed through cross-subsidy imposed on lower risk groups or met through some other mechanism. An alternative starting point could be to ensure that those facing the greatest possible handicap (however defined) from genetic conditions retain access on reasonable terms.

A multitude of other models could be proposed, each of which will embody their own trade-offs and give rise to different cost profiles and health outcomes. This will also give rise to a host of ethical issues. Should the treatment of genetic information be different from other personal characteristics that influence insurance premiums but over which the individual concerned has no control, such as sex and age? Is discrimination by insurance companies on the basis of genotype normatively the same as discrimination according to sex, the use of which as a rating factor the European Union prohibited in 2012 (Commission 2012)? Can and should there be separate ethical frameworks for different types of insurance?

## 7 Conclusion

Insurance is valuable. Well-functioning insurance markets help individuals manage the risk of adverse events whose timing and impact is uncertain. Risk disclosure and actuarial pricing of risk are fundamental to insurance underwriting. We considered how developments in genomics could result in unintended impacts on insurers and policy holders, with a particular focus on the regulatory context of the United Kingdom.

We contend that expansions in the volume and quality of genetic data, the blurring of diagnostic and predictive genetic testing, and new evidence on the association between genotype and mortality, healthcare costs and related phenotypes mean that conversations about their consequences and new regulatory developments are now needed. These issues affect both the specific regulatory regime prevailing in the United Kingdom (with the Code’s distinction between diagnostic and predictive tests) as well as more general issues regarding whether and to what extent deviations from actuarial pricing of genetic risks are justified. Both the UK-specific issues and the more general issues require deliberative processes to examine how new regulatory approaches relating to the use of genetic data might be developed.

Any new approaches will depend in a fundamental way on normative ideas of fairness. Deviations from actuarial pricing could increase the aggregate costs of insurance, but in doing so could reflect preferences regarding fairness in the accessibility and pricing of insurance that depends on genetic information. Future regulatory developments in this area will likely involve identifying relevant normative principles, quantifying these costs, and developing mechanisms for distribution of costs amongst individuals.

## Footnotes

1 We use “genetics” and “genomics” interchangeably throughout
